# Multisystemic Inflammatory Syndrome Temporally Associated with COVID-19 in a Regional Pediatric Hospital from México

**DOI:** 10.3390/pediatric15020030

**Published:** 2023-05-26

**Authors:** Joel Barroso-Santos, Angelina Ingrid Robledo-Martínez, Sara Elva Espinosa-Padilla, Rubén Genaro Hurtado del Ángel, Felipe Arteaga-García, Mónica Langarica-Bulos, José Antonio Madrid-Gómez-Tagle, Beatriz Adriana Sánchez-Reyes, Sarai Eunice Hernández-Cadena, Jorge Iván Suárez-Soto, Carolina Delgado-Amézquita, Brenda Godínez-Hernández, Octavio Otamendi-Canales, Angélica Saraí Jiménez-Osorio

**Affiliations:** 1 Hospitalization Service, Hospital del Niño DIF Hidalgo, Pachuca 42080, Hidalgo, Mexicomo.langarica@gmail.com (M.L.-B.);; 2 Immunodeficiency Research Unit, National Institute of Pediatrics, Coyoacán, Mexico City 04530, Mexico; 3 Immunology and Allergy Service, Club Pediatría, Pachuca 42080, Hidalgo, Mexico; 4 Área Académica de Enfermería, Instituto de Ciencias de la Salud, Universidad Autónoma del Estado de Hidalgo, San Agustín Tlaxiaca 42060, Hidalgo, Mexico; 5 Research Service, Hospital del Niño DIF Hidalgo, Pachuca 42080, Hidalgo, Mexico

**Keywords:** multisystemic inflammatory syndrome, COVID-19, children

## Abstract

Multisystemic inflammatory syndrome (MIS-C) is an inflammatory condition temporally associated with COVID-19 in children; nevertheless, the clinical and immunologic spectrum of MIS-C is heterogeneous, and its long-term effects are unknown. During the period of August 2020 to December 2021, a total of 52 MIS-C cases were confirmed in pediatric patients from the Hospital del Niño DIF Hidalgo, diagnosed using criteria from the World Health Organization. All patients had serologic IgG confirmation of SARS-CoV2, the mean age of the patients was 7 years, and 94% of the patients did not have a previous underlying disease. In addition to the presentation of lymphopenia, neutropenia, and thrombocytopenia, elevations in D-dimer and ferritin levels were observed in all patients. There was clinical improvement with intravenous gamma globulin and corticosteroid treatment.

## 1. Introduction

On 25 April 2020, the National Health Service of the United Kingdom issued a warning about a multisystemic inflammatory syndrome in children (MIS-C) that was temporally associated with SARS-CoV-2, with clinical characteristics similar to those found in Kawasaki disease (KD), toxic shock syndrome, hemophagocytic lymphohistiocytosis, and macrophage activation syndrome [1,2]. The first reports in 2020 compared MIS-C symptoms to those of KD (with fever mucocutaneous features and cardiac sequelae), but the recent Clinical Guidance established by the American College of Rheumatology (version 3) discusses differences between the MIS-C and KD phenotypes that are worth mentioning. First, this guide states that the incidence of KD is higher in Japan and East Asia, and it is common in children under 5 years of age. Additionally, the clinical presentations of left ventricular dysfunction and shock are more characteristic of patients with MIS-C than KD, and gastrointestinal and neurologic symptoms are more frequent in MIS-C patients [3].

The etiology of KD is far from fully understood, although it has been hypothesized that a respiratory infectious agent infecting the bronchial ciliated epithelium could be the causal agent of KD [4]. Luchman (2021) [5] proposed an analogy because in SARS-CoV-2 animal models, the neutralizing antibodies of SARS-CoV-2 protein S enhance the inflammatory response associated with MIS-C [6]. Therefore, a complex comprehension of this inflammatory phenomenon is required in order to understand the immune responses to SARS-CoV-2 in infants to establish an appropriate approach to MIS-C [7,8,9].

The World Health Organization (WHO) defines MIS-C as the presence of a fever lasting ≥3 days in children and teenagers from 0 to 19 years old and two of the following symptoms: rash or bilateral non-purulent conjunctivitis or signs of mucocutaneous inflammation (oral, hands, or feet), hypotension or shock, features of myocardial dysfunction, pericarditis, valvulitis or coronary anomalies (echo findings included or high troponin/NT-proBNP), evidence of coagulopathy, (prothrombin time or thromboplastin or high D-dimer), acute gastrointestinal issues (diarrhea, vomiting, or abdominal pain) and increased biomarkers of inflammation, such as the erythrocyte sedimentation rate (ESR), C-reactive protein (CRP), or procalcitonin (PCT), excluding a microbial causal agent of inflammation, such as bacterial sepsis, staphylococcus, or streptococcal shock syndrome, as well as evidence of COVID-19 (RT-PCR), such as antigen tests, positive serology, or probable contact with COVID-19 patients [10,11,12]. Even if the reports have increased during the pandemic, the description of the clinical presentations of MIS-C is imperative for the comprehension of this new syndrome and its approach in the pediatric population.

## 2. Materials and Methods

This was an observational, retrospective, and descriptive study including pediatric patients who attended the Hospital del Niño DIF Hidalgo (HNDH) in Mexico from August 2020 to December 2021. Since August 2020, the HNDH has been declared a COVID-19-hospital, and the HNDH–MIS-C Committee was established to identify pediatric patients with MIS-C according to the WHO criteria [13,14]. Therefore, at the operational level, this observational study includes pediatric patients with fever for more than three days, physical signs of inflammation at admission, clinical data for inflammatory biomarkers, and positivity for COVID-19 via a serologic test (IgG and IgM) or previous contact with COVID-19.

Clinical measures taken at admission included blood count, CRP, ESR, PCT, D-dimer, ferritin, creatinine, and sodium. We also registered demographic and anthropometric data upon admission. All patients who tested negative for COVID-19 or had a fever duration of less than 3 days were excluded. In order to evaluate a diagnosis of incomplete KD/KD shock syndrome, chest radiology and echocardiography were carried out at admission.

The patients’ parents or legal guardians signed the informed consent form, and all the cases were registered according to the Institutional Ethics Committee guidelines (register number CICEICB-2021-CC07).

### Statistical Analysis

The results of the continuous data (age, body mass index: BMI, CRP, PCT, ESR, D–dimer, ferritin, platelets, lymphocytes, and sodium) are presented as means ± standard deviations (SDs) for variables with a Gaussian distribution. In cases of non-Gaussian distribution, the results of continuous data are presented with the median (interquartile range: IQR). Frequencies are presented in numbers (percentages) for categorical data. We analyzed associations between the variables of interest (age, sex, PICU stay, and MIS-C severity). The analysis was performed using STATA v.14.

## 3. Results

Fifty-two patients met the case definition of MIS-C (5), with a mean duration of fever of 4 days. More cases of males diagnosed with MIS-C were included (33 males and 19 females, 63.4% vs. 36.6%, respectively), with a mean age of 7 years (Min: 1 and Max: 14 years) and 19 children (36.6%) under 5 years of age. The median BMI was 16.6 (IQR = 15.4 − 17.6) kg/m^2^. Of the participants, 7.7% (n = 4) were in the obese range, 23.1% (n = 12) were overweight, and 7.7% (n = 4) were underweight. All patients were positive for SARS-CoV-2, determined via IgG serologic test and 41.4% via the epidemiologic nexus. PCR confirmation was accessible in two patients only, both of whom were positive. The most frequent symptoms identified at admission to the hospital were conjunctivitis, cutaneous rash, emesis, oral changes, abdominal pain, and diarrhea. Irritability was the most common sign of mental alteration (Table 1).

CRP, PCT, ESR, D-dimer, and ferritin concentrations were determined at admission, and the median values of the results were superior to the maximum reference limits (Table 2). The serum CRP concentrations were found to be in the normal range (<5 mg/dL) in seven patients (13.4%), and the PCT levels were under 0.1 ng/mL in only three (5.7%) patients. On the other hand, the median for platelet values was found to be in the normal range (184 × 10^9^ cells/L), and the platelet values were below the lower limit (150 × 10^9^ cells/L) in only 18 patients (34.6%). Lymphopenia was present in 37 patients (71.1%). In 17 patients (32.7%), serum sodium levels were found to be below the lower limit (<135 mEq/L), although the average was in the normal range.

The mean hospital stay was 6 days (min: 2, max: 25). During their stay in the hospital’s Pediatric Intensive Care Unit (PICU), 23.1% of the patients manifested shock criteria, and 1 case manifested moderate upper gastrointestinal bleeding as a complication, thus requiring aminergic management for 3 days in the PICU. Of the patients, 15.4% (n = 8) presented with criteria for acute kidney injury according to the RIFLE criteria (risk, injury, failure, loss, and end stage), defined as a 50% increase in serum creatinine at 24 h [15]. Vasopressor support was applied in nine cases (17%, Table 3).

We observed bilateral opacities in eight patients (15.4%). The echocardiogram evidenced three patients with pericarditis, and one of them demonstrated left ventricular function decline, which was recovered after treatment. Respiratory failure was observed in 11 patients (21%), and 7 patients required noninvasive mechanical ventilation, oxygen masks (n = 8), and nasal tips (n = 1) (Table 3).

Fifty-one cases (98%) were treated with gamma globulin (2 g/kg of weight/day for 12 h, with a maximum dose of 80 gm), 96.1% (n = 50) with methylprednisolone (2 mg/kg of weight/day for 14 days), and 88% with acetylsalicylic acid (50 mg/kg of weight/day for 8 weeks until a follow-up echocardiogram without alterations). Prior to hospital admission, four patients (7.7%) received antiviral treatment (two with acyclovir and two with oseltamivir), and 53.8% (n = 28) received empirical antibiotic treatment for at least 48 h (Table 3). In the correlation analysis, no significant associations were observed when analyzing the frequency of the characteristics at admission according to age, sex, admission to the PICU, or MIS-C severity due to our small sample size.

Of the patients, 88.2% (n = 46) were discharged in a mean time of 8 days without clinical data indicating complications. However, 3 patients were discharged after 11 days due to the severity of MIS-C related to the PICU stay, the use of vasopressors, and respiratory support. Three patients had the longest hospital stays (15 to 25 days), which were related to underlying diseases. A severe MIS-C case was identified in a female patient who was diagnosed with high-risk myeloid lymphoblastic leukemia in 2017, aged 10 years, with an isolated relapse in the second-line protocol who was admitted to the PICU in February 2021 for febrile neutropenia and respiratory distress (intercostal indrawing and polypnea) with severe thrombocytopenia and elevated acute phase reactants (PCR = 3.8 mg/dL). After IVIG and methylprednisolone treatment, she was discharged on day 23 with a normal echocardiogram.

Mortality (n = 2) was observed in two patients. One male child with Down Syndrome (8 years old) was diagnosed with pre-B acute lymphoblastic leukemia in April 2019. On October 2020, he was admitted due to neutropenia and cervical abscess, with a history of COVID-19 infection 4 weeks prior to admission, which was confirmed by PCR. At admission, he presented with a fever of more than 3 days of evolution and elevated D-dimer (2000 mg/mL) without data indicating bacteremia. Chest tomography reported pneumonia with focal lobar interstitial condensation lesions which was predominantly bilateral at baseline. During the hospital stay, he was clinically stable with supplemental oxygen support. Intravenous gamma globulin (1 gr/kg/dose; 20 gr total) and methylprednisolone (2 mg/kg/day for 14 days) were administered. He was discharged on day 25 with a normal echocardiogram. One week later, this patient was readmitted for septic shock and died. The other case was a one-year-old female patient with congenital cardiopathy, post-operation for patent ductus arteriosus, and a history of perinatal asphyxia that required hospitalization for one month. She had a positive antigen test for SARS-CoV-2 with torpid evolution, septic shock, and cardiogenic shock which required broad-spectrum antimicrobial and antifungal management as well as management with vasoactive amines. After 25 days, hemodynamic and respiratory deterioration was observed without improvement, presenting septic and cardiogenic shock and death.

## 4. Discussion

In this report, we present the clinical characteristics of children with MIS-C associated with SARS-CoV-2 and with an exacerbated inflammatory response. The patients had heterogeneous phenotypes, and the differences in the first series of MIS-C cases in 2020 were reported as positivity for SARS-CoV-2, age of presentation, proportion of males, and less cardiac involvement.

The majority of patients were found to be positive for SARS-CoV-2 via serologic testing, which has been observed with less frequency in previous studies (20–53%) [16,17]. All patients with MIS-C presented with a fever and more than three criteria for MIS-C. The majority of cases reported correspond to males, with a proportion of 1.5 to 1 (male/female), as previously reported in the general population [18,19]. A higher prevalence of MIS-C in males has been reported in the Mexican population; although the reason is not clear, it could be due to a higher risk of developing COVID-19, and it has been hypothesized that specific immune defects could predispose males to MIS-C [20,21].

It has been suggested that KD and MIS-C are two pathological entities [22]. The age of presentation with MIS-C is older than in classic KD (10 years vs. 5 years in KD). Therefore, the age of presentation has been suggested as an important characteristic of MIS-C; nevertheless, given the epidemiological curse of the pandemic, children with MIS-C under one year of age have been documented in case series [23], coinciding with this report, which includes patients from August 2020 to July 2021, and with the severity of the clinical picture. Therefore, it is imperative to study the influence of SARS-CoV-2 variants to support the understanding of the pathophysiological mechanisms of MIS-C and its differences from KD.

The mechanisms that exacerbate the inflammatory response via SARS-CoV-2 are still a matter of investigation, even though current reports highlight important clinical differences. In a meta-analysis of 969 patients with MIS-C, low levels of leukocytes, platelets, and serum sodium were observed, including higher CRP, D-dimer, ferritin, and creatinine levels than in KD or incomplete KD patients, even though the PCT and ESR levels were similar [24,25]. In this series of cases, all patients showed higher levels of CRP, PCT, and D-dimer at admission, in addition to leukopenia.

Cardiovascular complications are a prominent characteristic of KD and are compatible with MIS-C, such as ventricular and valvular dysfunction or persistent coronary aneurysm, which is described in 20–25% of patients who do not receive immunomodulatory treatment [26,27,28]. Left ventricle systolic dysfunction has been described as the most frequent cardiac affection in a large proportion of children diagnosed with MIS-C [29,30]. In the first series of MIS-C cases described in the United Kingdom, cardiac dysfunction was present in 6/8 patients (75%). A subgroup of patients with an MIS-C diagnosis may present hypotension and shock via acute myocardial dysfunction, as well as hyperinflammation/systemic vasodilation [31,32]. The late development of the aneurysm in the coronary artery highlights the necessity of the continuous follow-up of these patients. In this report, we did not observe cardiovascular warning signs; the echocardiogram evidenced three patients with pericarditis, and only one of them had a decrease in left ventricular function, which recovered after immunomodulatory treatment.

The basis of the treatment for MIS-C is focused on immunomodulatory therapy with intravenous human gamma globulin, high, anti-inflammatory doses of corticosteroids, and their intensification in refractory cases with methylprednisolone pulse [33]. All patients who did not have an underlying disease showed a fast recovery with the indicated treatment. However, the presence of underlying diagnoses of acute lymphoblastic leukemia and myeloid lymphoblastic leukemia in two patients with leukopenia and trisomy 21 could be a bad prognosis factor, as has been previously reported [12].

## 5. Conclusions

D-dimer and PCT were present in all cases; however, cardiovascular complications were less frequent. The continuous communication of the clinical manifestations of MIS-C is important to increase the knowledge of this pathology; therefore, it the continuous follow-up of patients is imperative in order to know the possible mid- to long-term implications.

## Figures and Tables

**Table 1:** Summary of symptoms at hospital admission.

Symptoms	Frequency (%)
Duration of fever (days)	4 (min. 3, max. 5)
Diarrhea	30 (57.7)
Abdominal pain	40 (76.9)
Emesis	36 (69.2)
Cutaneous rash	38 (73)
Conjunctivitis	39 (75)
Cleft lips/strawberry tongue	35 (67.3)
Lymphadenopathy	26 (50)
Limb edema	28 (54.9)
Headache	15 (28.8)
Mental alteration/Irritability	15 (28.8)
Somnolence	8 (15.4)

**Table 2:** Laboratory test results from admission.

Test	Value	Reference Range
C-Reactive protein (mg/dL)	18.1 (6.3–26.5)	0–5 mg/L
Procalcitonin (ng/mL)	2.7 (0.8–9)	<0.1 ng/mL
Erythrocyte sedimentation rate (mm/h)	40 ± 15.3	0–10 mm/h
D-dimer	2974 (1457.7–6074)	<0.5 mg/mL
Ferritin	245.7 (175.7–463)	10–200 ng/mL
Platelets	184 (104.5–303)	150–400 × 10^9^ cells/L
Lymphocytes	1230 (907–2943)	3–9.5 × 10^3^ cells/μL
Sodium	135 ± 4.9	(135–145 mEq/L)

**Table 3:** Clinical characteristics of MIS-C patients during hospital stay.

Outcomes	Frequency (%)
General findings in PICU	
Shock	12 (23.1)
Gastrointestinal Bleeding	1 (1.9)
Acute Kidney Injury	8 (15.4)
Vasopressor support	
Norepinephrine	4 (7.7)
Epinephrine	1 (1.9)
Dobutamine	1 (1.9)
Norepinephrine + Epinephrine	2 (3.8)
Norepinefrine + Dobutamine	1 (1.9)
Cardiovascular findings	
Bilateral opacities	8 (15.4)
Acute pericarditis	4 (7.7)
Pericardia Effusion	3 (5.8)
LV ejection fraction <50%	1 (1.9)
Respiratory support	
Mechanic ventilation	7 (13.46)
Oxygen	3 (5.8)
Nasal tips	1 (1.92)
Pharmacologic therapy	
Empirical antibiotic treatment	28 (53.8)
Ceftriaxone	26 (50)
Clindamycin	8 (15.4)
Cefepime	2 (3.85)
Intravenous immunoglobulin	51 (98)
Methyl prednisolone	50 (96.15)
Acetylsalicylic acid	46 (88.4)
Antiviral treatment	4 (7.7)
Oseltamivir	2 (3.9)
Acyclovir	2 (3.9)
Mortality	2 (3.8)

## Data Availability

The data presented in this study are available upon request from the corresponding author. The data are not publicly available due to Hospital Privacy Statements.
